# Comparing microsatellites and single nucleotide polymorphisms to evaluate genetic structure and diversity in wolverines (*Gulo gulo*) across Alaska and western Canada

**DOI:** 10.1093/jmammal/gyae151

**Published:** 2025-01-15

**Authors:** Elise M Stacy, Martin D Robards, Thomas S Jung, Piia M Kukka, Jack Sullivan, Paul A Hohenlohe, Lisette P Waits

**Affiliations:** Environmental Science Program, University of Idaho, 875 Perimeter Drive MS 1136, Moscow, ID 83844-1139, United States; Wildlife Conservation Society Arctic Beringia, 302 Cushman Street, Suite 203, Fairbanks, AK 99701, United States; Department of Environment Government of Yukon, 419 Range Road, Whitehorse, YT Y1A3N1, Canada; Department of Renewable Resources, University of Alberta, 751 General Services Building, 9007 - 116 St NW, Edmonton, AB T6G 2E3, Canada; Department of Environment Government of Yukon, 419 Range Road, Whitehorse, YT Y1A3N1, Canada; Department of Biological Sciences, University of Idaho, 875 Perimeter MS 3051, Moscow, ID 83844-3051, United States; Department of Biological Sciences, University of Idaho, 875 Perimeter MS 3051, Moscow, ID 83844-3051, United States; Department of Fish and Wildlife Sciences, University of Idaho, 875 Perimeter Drive MS 1136, Moscow, ID 83844-1136, United States

**Keywords:** isolation by distance, mustelid conservation, RAD sequencing, spatial autocorrelation

## Abstract

The Wolverine (Gulo gulo) is a cold-adapted species of conservation interest because it is sensitive to human development, disturbance, exploitation, and climate warming. Wolverine populations have been studied across much of their distributional range to evaluate patterns of genetic diversity, genetic structure, and gene flow. Little population structure has been detected in northwestern North America with microsatellite loci, but low genomic diversity in wolverines may limit detection of genetic differences in this highly vagile species. Here, we genotyped a relatively large sample of wolverines from across Alaska (US) and adjacent Yukon (Canada) with 12 microsatellite loci (*n *= 501) and 4,222 single nucleotide polymorphisms (SNPs; *n* = 201) identified using restriction-site associated DNA sequencing. We compared the relative ability of our microsatellite and SNP datasets to evaluate population genetic structure, genetic diversity, differentiation, and isolation by distance (IBD). We predicted that the SNP dataset would detect a higher degree of genetic structure and provide more significant support for IBD. We found evidence for multiple genetic clusters, including genetic distinctiveness of wolverines in southeast Alaska and on the Kenai Peninsula. The SNP dataset detected additional genetic clusters that align largely with ecoregions, and the SNP dataset showed stronger evidence of IBD, while the 2 datasets were generally consistent in estimates of genetic diversity and differentiation among regional groups. Our results highlight the importance of genomic methods to assess gene flow in wolverines. Identifying population genetic structure allows an assessment of the potential impacts of conservation threats and is an important precursor for designing population monitoring programs.

## Teaser

Increasing the number and density of genetic loci has increased the resolution of population structure analysis in wide-ranging mammal species. This study assesses genetic population structure, isolation by distance (IBD), and genetic diversity for the North American Wolverine across their continuous northwestern range with 12 microsatellite loci and 4,222 single nucleotide polymorphisms (SNPs), finding that SNPs have more power to describe population structure and IBD. Population groups identified with SNPs largely align with ecoregions and geographic features, and we recommend monitoring efforts on populations with lower genetic diversity.

Species in the family Carnivora are of global conservation concern because they have higher levels of vulnerability and endangerment than most mammals (Ripple et al. 2014; Marneweck et al. 2021). The leading causes of decline are human persecution, habitat loss, and habitat fragmentation (Marneweck et al. 2021). Evaluating levels of gene flow, genetic structure, and genetic diversity within carnivore populations can provide valuable information for conservation and management. Defining population structure and levels of isolation can inform management unit designation for harvest regulation of bobcats (*Lynx rufus*) in South Dakota (Fetherston et al. 2024) and has confirmed separate management units between Central European and Baltic wolf (*Canis lupus*) populations (Szewczyk et al. 2019). Defining population structure and levels of isolation is also an important precursor to identifying populations in need of monitoring programs (Allendorf et al. 2010).

One globally vulnerable carnivore is the Wolverine (*Gulo gulo*), the largest terrestrial member of the family Mustelidae. The Wolverine is a highly vagile, cold-adapted species with a circumpolar distribution (Fisher et al. 2022). In North America, Wolverine distribution and population connectivity have been shaped by anthropogenic disturbances, climate, and natural landscape features (Aubry et al. 2007; Copeland et al. 2010). Vulnerability to climate change and human disturbance have prompted increased conservation concern for populations at the southern periphery of their North American range in Wyoming, Montana, Idaho, and Washington (Supplementary Data SD1). Thus, wolverines across the contiguous United States have been designated as a distinct population segment that was recently listed as threatened under the US Endangered Species Act (ESA; Fish and Wildlife Service 2023). Wolverines are also listed as a species of Special Concern under Canada’s Species at Risk Act and are protected from harvest in Ontario (Fisher et al. 2022), and monitoring has informed the reduction of harvest quotas for populations in southwestern Canada (Mowat et al. 2020).

At the southern periphery of their range in North America, wolverines are confined to alpine and montane habitats (Aubry et al. 2007; Schepens et al. 2023), which are fragmented by roads, human development, and warmer low-elevation regions. Wolverine populations in southern Canada and the contiguous United States are more isolated and have lower levels of genetic diversity and less gene flow between regions than their northern counterparts (Kyle and Strobeck 2001, 2002; Cegelski et al. 2003, 2006). Studies investigating genetic connectivity in relation to landscape features in their southern range documented positive associations between gene flow and snow and terrain ruggedness (Schwartz et al. 2009; Balkenhol et al. 2020) and negative associations with anthropogenic features (Sawaya et al. 2019; Balkenhol et al. 2020). These results led to increased support for wildlife overpasses in Canada parks and were cited in the justification for ESA listing of the contiguous US distinct population segment (Fish and Wildlife Service 2023).

Conservation and management focus on wolverines in their northern range differs from the southern range because wolverines are a harvested furbearer of cultural significance in Alaska and northern Canada (Bonamy et al. 2019). Harvest refugia are likely important for sustaining Wolverine populations in quota-free management areas (Kukka et al. 2022) and limits on trapping only exist near major human population centers in Alaska where harvest pressure is highest (Golden et al. 2007). There is little conservation attention towards wolverines in their northern range, but the increasing rate of development due to natural resource extraction and accelerated Arctic warming due to climate change will likely negatively impact northern Wolverine populations (Glass et al. 2022). Additionally, northern populations of wolverines are an important source of dispersers that likely maintain genetic diversity for southern populations (Cegelski et al. 2006). Therefore, describing and monitoring Wolverine connectivity and genetic diversity in their northern range serves an important role in informing management and conservation strategies for the species across North America.

Previous genetic analyses of Wolverine populations in North America have employed mitochondrial DNA sequences and nuclear DNA microsatellite loci (Wilson et al. 2000; Kyle and Strobeck 2001, 2002; Tomasik and Cook 2005; Cegelski et al. 2006; Zigouris et al. 2013; Krejsa et al. 2021). These studies detected minimal population structure and high gene flow across Alaska and the Yukon, where populations mostly appear to be panmictic outside of relatively isolated geographic regions such as the Kenai Peninsula and Southeast Alaska. However, our ability to detect population structure in regions with higher gene flow may be limited. Wolverines occupy large home ranges between 73 and 1,506 km^2^ and can disperse over 1,000 km (Magoun 1985; Whitman et al. 1986; Copeland 1996; Landa et al. 1998; Vangen et al. 2001; Persson et al. 2010; Packila et al. 2017). Therefore, large spatial distributions need to be sampled to detect population structure. Additionally, whole genome analysis of wolverines from Scandinavia and northern Canada reveals they have low genomic diversity relative to wolves (*Canis lupus*) and grizzly bears (*Ursus arctos*; Ekblom et al. 2018; Lok et al. 2022) and microsatellite loci describe less variation in Scandinavian wolverines compared to Scandinavian wolves (Väli et al. 2008). This pattern could be due to their polygamous mating structure and low fecundity leading to a low effective population size relative to other species because life history traits are correlated with genetic diversity (Romiguier et al. 2014; Ellegren and Galtier 2016).

More loci may be needed to detect population structure in wolverines compared to other vagile carnivores with higher genome diversity. Large SNP datasets have provided increased resolution for delineating population structure when compared to microsatellites in a variety of species (Sunde et al. 2020), notably for species with large home ranges and high dispersal capabilities, where microsatellite datasets lacked power to detect population structure (Malenfant et al. 2015; Lah et al. 2016; Puckett and Eggert 2016). Thus, the greater resolution of large SNP datasets may be particularly insightful in illuminating genetic structure of wolverines. In fact, long-term monitoring of wolverines in Scandinavia has transitioned from microsatellites to SNPs for increased power to evaluate genetic diversity, gene flow, relatedness, and parentage (Ekblom et al. 2018, 2021; Lansink et al. 2022).

Subtle, and previously undetected, population structure may be present among wolverines in their northern North American range. Wolverines occupy a variety of ecoregions across their northwestern distribution including Arctic, tundra, taiga, boreal forest, and coastal ecoregions (Level 2 ecoregion designations; US Environmental Protection Agency 2010). These habitats are characterized by differences in temperature and precipitation, as well as available Wolverine den site structures and prey (Magoun 1985; Whitman et al. 1986; Lewis and Flynn 2006; Magoun et al. 2017; Glass 2022), which may manifest as local adaptations among populations. Field studies and genetic data document wolverines exhibiting sex-biased dispersal (Vangen et al. 2001; Cegelski et al. 2003; Chappell et al. 2004; Aronsson and Persson 2018; Copeland et al. 2018), where females tend to occupy territories near their natal territory for longer than males. Additionally, subadult wolverines can stay in their natal territory spending time with both parents (Copeland et al. 2018). Sex-biased dispersal and parental rearing could drive natal-habitat-biased dispersal, where individuals are more likely to stay and breed in familiar habitat, which could create genetic structure among wolverines under isolation by environment model as has been documented in other carnivores (Sacks et al. 2004; Sanz-Pérez et al. 2018).

The aims of our study were to: (i) evaluate genetic variation, population structure, and isolation by distance (IBD) from a comprehensive geographic sampling of wolverines in Alaska and the Yukon using 12 nuclear DNA microsatellite loci; (ii) evaluate the same genetic parameters from a subset of Wolverine samples spanning the same geographic range using thousands of SNP loci; (iii) compare results between the microsatellite and SNP datasets; and (iv) use the genetic data to identify regional population groups, inform management, and identify potential genetic threats for wolverines across Alaska and the Yukon. We predicted that the SNP dataset would have more power to detect population structure in this low genomic diversity and highly vagile species, where subtle population structure may be caused by natal-habitat-biased dispersal. Comparison between the microsatellite and SNP datasets will test the utility of these methods for the genetic assessment of vagile, low-density species and provide an updated assessment of Wolverine gene flow and genetic structure in the core of their range in North America.

## Methods

**Fig. 1.**
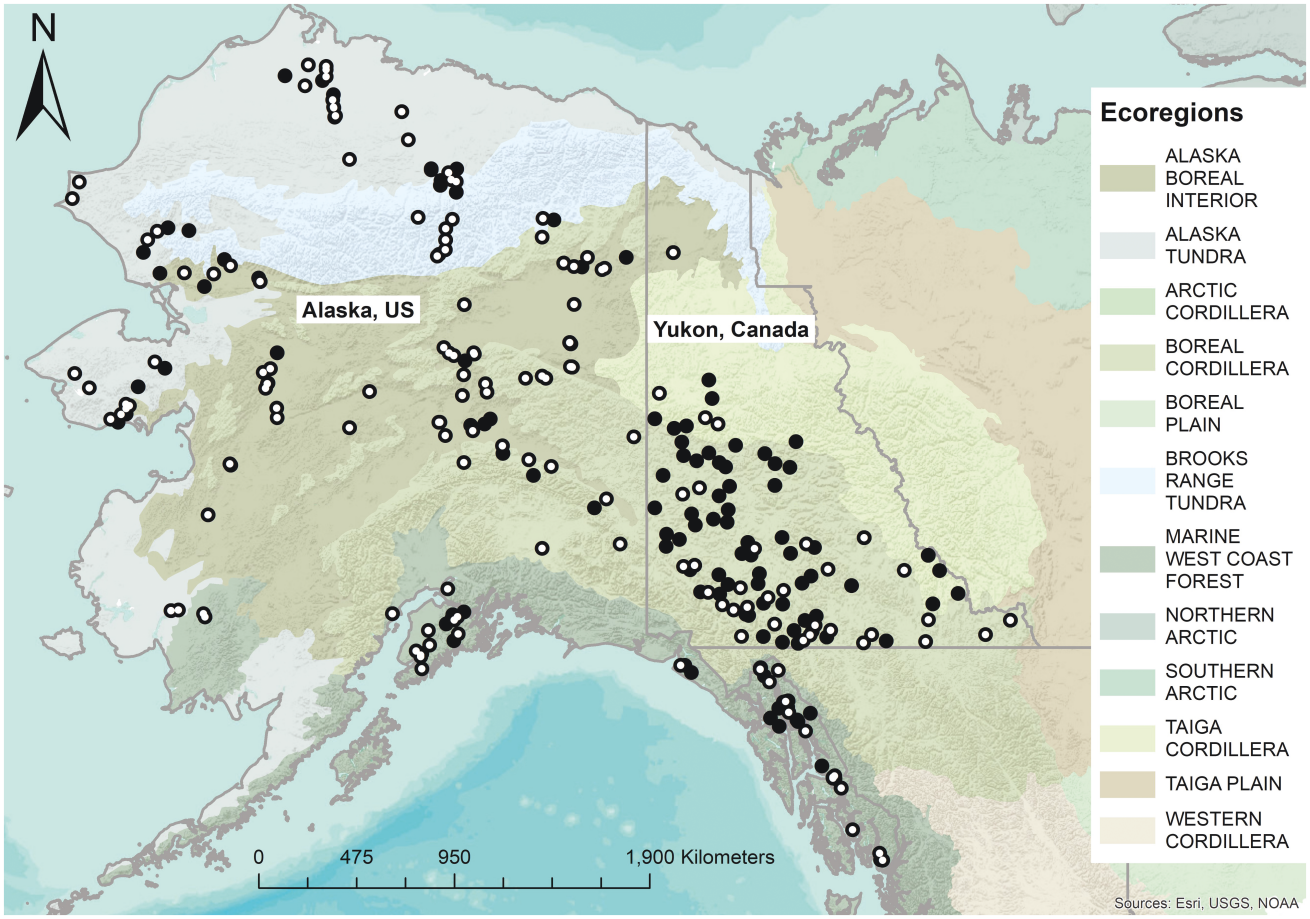
Wolverine (*Gulo gulo*) sample distribution across Alaska and the Yukon. Open circles represent samples that have been genotyped with both microsatellite loci and SNPs, while filled circles represent samples that have only been genotyped with microsatellite loci. Level 2 ecoregion designations for North America (U.S. Environmental Protection Agency 2010) are displayed in color, and political boundary lines are in dark gray.

We obtained Wolverine samples through voluntarily submissions by individual trappers, fur handlers, the University of Alaska Museum of the North (Supplementary Data SD2). Tissue samples (*n* = 597) originated from harvested wolverines (e.g., Jung et al. 2016, 2020), whereas ear punches (*n* = 10) and hair (*n* = 7) originated from a study in which wolverines were live trapped (Glass 2022). Samples were collected across Alaska (US), and Yukon (Canada) from 2000 to 2020 (Fig. 1). Alaskan samples were georeferenced either with GPS coordinates or a description of nearest landmarks to trapping location, while a handful only had broader sampling information to nearest town. Those from the Yukon had GPS coordinates from the geometric center of the Registered Trapping Concession where the Wolverine was harvested (Kukka et al. 2022).

DNA was extracted using Qiagen DNeasy tissue kits and standard extraction protocols and treated with RNase A (Qiagen). Wolverines were genotyped in duplicate at 12 polymorphic microsatellite loci: Gg454, Gg443, Gg452, Gg465 (Walker et al. 2001); Gg42-1, Gg192-1, Gg37-2 (Walker et al. 2001; redesigned Krejsa et al. 2021); Gg3, Gg14, Tt-4 (Davis and Strobeck 1998); Gg-7-1 (Davis and Strobeck 1998; redesigned Krejsa et al. 2021); Ggu216 (Duffy et al. 1998) in a single PCR multiplex with DNA extraction and PCR negative controls (PCR profile in Supplementary Data SD3). Only microsatellite locus genotypes that were identical in both replicates were included in the dataset.

We selected a subset of the microsatellite genotyped samples for restriction-site associated DNA sequencing (RAD seq) based on geographic distribution, DNA fragmentation, and DNA concentrations ≥ 5 ng/µl. We quantified fragmentation via agarose gel electrophoresis, and we quantified DNA concentration using the Qubit double-stranded DNA High Sensitivity Assay Kit (Thermo Fisher Scientific Inc., Waltham, MA, USA). We followed the RAD seq protocol outlined in Ali et al. (2016), excluding the targeted bait capture with modification to use biotinylated adapters (a protocol referred to as “bestRAD”). We prepared 4 libraries using the *sbfI* restriction enzyme and sequenced each library on Illumina HiSeq 4000 and NovaSeq with 150 base pair paired-end reads. Samples were duplicated (~10% per DNA library) to calculate a genotype mismatch rate (Mastretta-Yanes et al. 2015).

We processed and quality filtered RAD sequence reads using the Research Computing and Data Services (RCDS) computational infrastructure of the Institute for Integrative Data Sciences (IIDS) at the University of Idaho. We used STACKS version 2.6 (Rochette et al. 2019) to demultiplex sequence reads, and we aligned our reads to the North American Wolverine genome (Lok et al. 2022; GCA_024510155.1) using Bowtie2 version 2.2.9 (Langmead and Salzberg 2012). We performed variant calling with the STACKS gstacks and populations functions, specifying remove PCR duplicates (--rm-pcr-duplicates) and all samples as 1 population. Subsequently, we used VCFTOOLS version 0.1.16 for quality filtering. We filtered for genotypes with ≥3 and ≤60 read depth, then following an iterative approach similar to O’Leary et al. (2018) to maximize sample retention; we alternated 5 times between filtering for per locus missingness (thresholds 0.7 to 0.85) and individual missingness (thresholds 0.99 to 0.6). We thinned loci to 100,000 base pairs apart to minimize linkage, and filtered loci for a minor allele count of 3 (minor allele frequency of 0.007). To achieve a putatively neutral locus dataset for population genetics analyses, we removed loci with evidence of being under selection (i.e., outlier loci). Outlier loci were detected using R packages “*pcadapt*” (Luu et al. 2017; Privé et al. 2020), “*tess3r*” (Caye et al. 2016), and “*LEA*” (Frichot and François 2015) using Benjamini–Hochberg’s algorithm with a false detection rate of 0.05 (Supplementary Data SD3), and outliers detected by any of the methods were removed (R Core Team 2024). To remove potential paralogous loci, we removed loci with an observed heterozygosity greater than 0.5.

Due to the potential impact of close relatives in downstream analyses (Rodríguez-Ramilo and Wang 2012), relatedness was calculated for the SNP dataset using R package “*related*” using the Wang measure of relatedness (Wang 2002, Pew et al. 2015). Individuals with a relatedness value ≥ 0.49 were removed. Due to low power available to calculate relatedness with the microsatellite dataset, maximum-likelihood relatedness was calculated (Kalinowski et al. 2006), and individuals with ≥ 0.69 relatedness values were removed along with those identified as related using the SNP dataset. Subsequent population genetics analyses were performed on the microsatellite dataset and SNP dataset. When direct sample set comparisons were needed to remove the effect of sample distribution and sample size, the microsatellite dataset was subset to only include the same individuals as the SNP dataset.

### Analysis

For both the microsatellite and SNP datasets, population structure was assessed with the Bayesian clustering analysis program STRUCTURE (Pritchard et al. 2000). The program was run under the admixture model with correlated allele frequencies, and with 100,000 burn-in followed by 1,000,000 Markov Chain Monte Carlo replication steps for *K* = 1 to 10 with 20 replicates per *K*. The most likely number of clusters (*K*) was evaluated using the likelihood method and delta *K* method (Evanno et al. 2005), calculated and visualized with R package “*pophelper*” (Francis 2017). To analyze population structure with a nonmodel-based method, principal component analysis (PCA) was performed for both datasets using R package “*adegenet*” (Jombart and Bateman 2008).

Regional groupings of the samples were defined after our population structure analysis and were informed by population structure results, geographic features, and ecoregions. Individual genetic assignments from program STRUCTURE (Pritchard et al. 2000) for both the SNP and microsatellite datasets were used to group individuals with majority population assignment or similar patterns of admixture that fit within geographic boundaries. Geographic boundaries such as mountain ranges, relatively isolated areas (e.g., Kenai Peninsula), and ecoregions (Level 2 ecoregion designations; U.S. Environmental Protection Agency 2010) were used to further inform our demarcation of putative regions. These regional definitions were used to organize samples for visualization of population structure figures and for calculating regional differentiation and genetic diversity.

Microsatellite loci were checked for Hardy–Weinberg equilibrium within regional groups and linkage disequilibrium across the total dataset with R package “*genepop*” (Rousset 2008), and we controlled for false discovery when comparing multiple tests with the Benjamini–Hochberg’s algorithm with a false detection rate of 0.05. Genetic differentiation and diversity metrics were calculated for both datasets and grouped by defined regions. Pairwise *F*_*ST*_ (Weir and Cockerham 1984) was calculated between regional groupings with R package “*Hierfstat*” (Goudet 2005), and statistical significance was evaluated with the boot.ppbetas function, with 999 bootstraps. Expected heterozygosity (*H*_*E*_) was calculated in the R package “*adegenet*” (Jombart and Bateman 2008) for both datasets. We tested whether there were significant differences in *H*_*E*_ between each region and the total study area with Hs.test function using 499 permutations, and set our alpha corrected for multiple testing with a Bonferroni correction.

Individual level pairwise genetic distance was calculated for wolverines with detailed location information in the microsatellite (*n* = 492) and SNP (*n* = 201) datasets. To investigate sample distribution and size effects, genetic distance was also calculated on the subset of microsatellite data (*n* = 201) that included samples with SNP genotypes. The dist function in the R package “*stats*” was used to calculate Euclidean genetic distances between individuals, which excluded missing data values from the calculation and scaled the pairwise distance values by the amount of missing data. To compare genetic distance estimates calculated from the microsatellite and SNP data, Euclidean genetic distance estimates resulting from the SNP and subset microsatellite datasets were directly compared via a Mantel test conducted in R package “*vegan*” (Oksanen et al. 2022) using the Pearson method (Rodgers and Nicewander 1988) and 9,999 permutations.

We assessed IBD by estimating the relationship between pairwise geographic distance (geodesic distance) calculated in R package “*Geosphere*” (Hijmans 2017) and pairwise Euclidean genetic distances via Mantel tests in R package “*vegan*” (Oksanen et al. 2022), again using the Pearson method and 9,999 permutations. We also assessed the scale and strength of IBD across distance classes via Mantel correlograms in R package “*vegan*” (Oksanen et al. 2022) with 9,999 permutations and Sturges’ rule (Sturges 1926) for binning distance classes.

## Results

### Datasets

A total of 614 wolverines were genotyped at 12 multi-allelic microsatellite loci, and 540 samples successfully amplified resulting in 78 alleles ranging from 4 to 11 alleles (mean of 6.5) per locus. We calculated an average of 1.1% missing alleles across all individuals and loci in the microsatellite dataset. Missing data was due to unamplified loci in both replicates, mismatches between replicates due to unamplified loci, or mismatches due to different allele calls. There was < 1% mismatch rate between replicates. No microsatellite loci showed evidence of linkage disequilibrium, and one locus was out of HWE in one region (Supplementary Data SD4 and SD5). A total of 283 wolverines were RAD sequenced, and 220 individuals passed individual missingness thresholds and were SNP genotyped. We removed 220 outlier loci and 37 loci with heterozygosity greater than 0.5, and we retained 4,222 bi-allelic loci resulting in 8,444 alleles. Our SNP dataset had an average missing data of 9.5% and < 2% allele mismatch rate between replicate samples. After filtering to remove related individuals, 501 individuals (*n* = 39 related removed) were retained from the microsatellite dataset, and 201 (*n* = 19 related removed) individuals were retained from the SNP dataset. The distribution of relatedness values can be viewed in Supplementary Data (SD15). Results for population structure, diversity, and differentiation are presented for the entire microsatellite dataset (*n* = 501) and SNP dataset (*n* = 201). Results for IBD are presented for the entire microsatellite dataset, which had detailed location information (*n* = 492), the subset microsatellite dataset, which only includes samples with SNP genotypes (*n* = 201), and SNP dataset (*n* = 201).

**Fig. 2.**
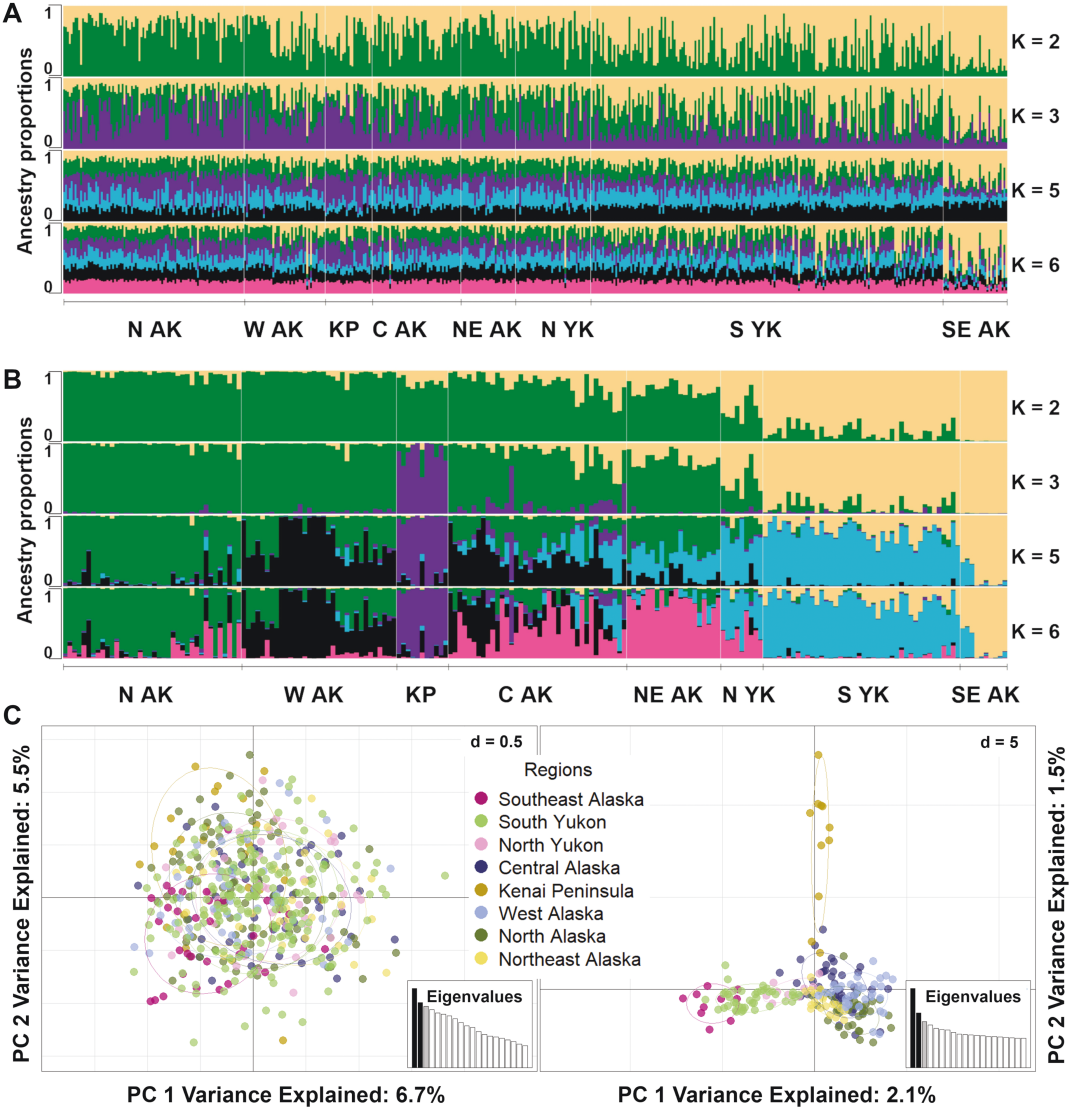
Alaska and Yukon Wolverine (*Gulo gulo*) genetic structure plots from program STRUCTURE a) and b) and PCA c) for SNP and microsatellite datasets. STRUCTURE Q-value plots showing the proportion of each wolverine’s ancestry being assigned to different *K* values (i.e., genetic clusters) for 501 microsatellite genotyped samples a) and 201 SNP genotyped samples b). Each individual bar represents an individual Wolverine. Displayed are all statistically supported *K* values for both datasets, where *K* = 3 was supported for the microsatellite dataset, and *K* = 2 and 6 were supported for the SNP dataset. From left to right, samples are organized by region starting with North Alaska (N AK), West Alaska (W AK), Kenai Peninsula (KP), Central Alaska (C AK), Northeast Alaska (NE AK), North Yukon (N YK), South Yukon (S YK), and Southeast Alaska (SE AK). PCA c) shows results for the microsatellite dataset (left) and SNP dataset (right), with individuals color coded by region.

**Fig. 3.**
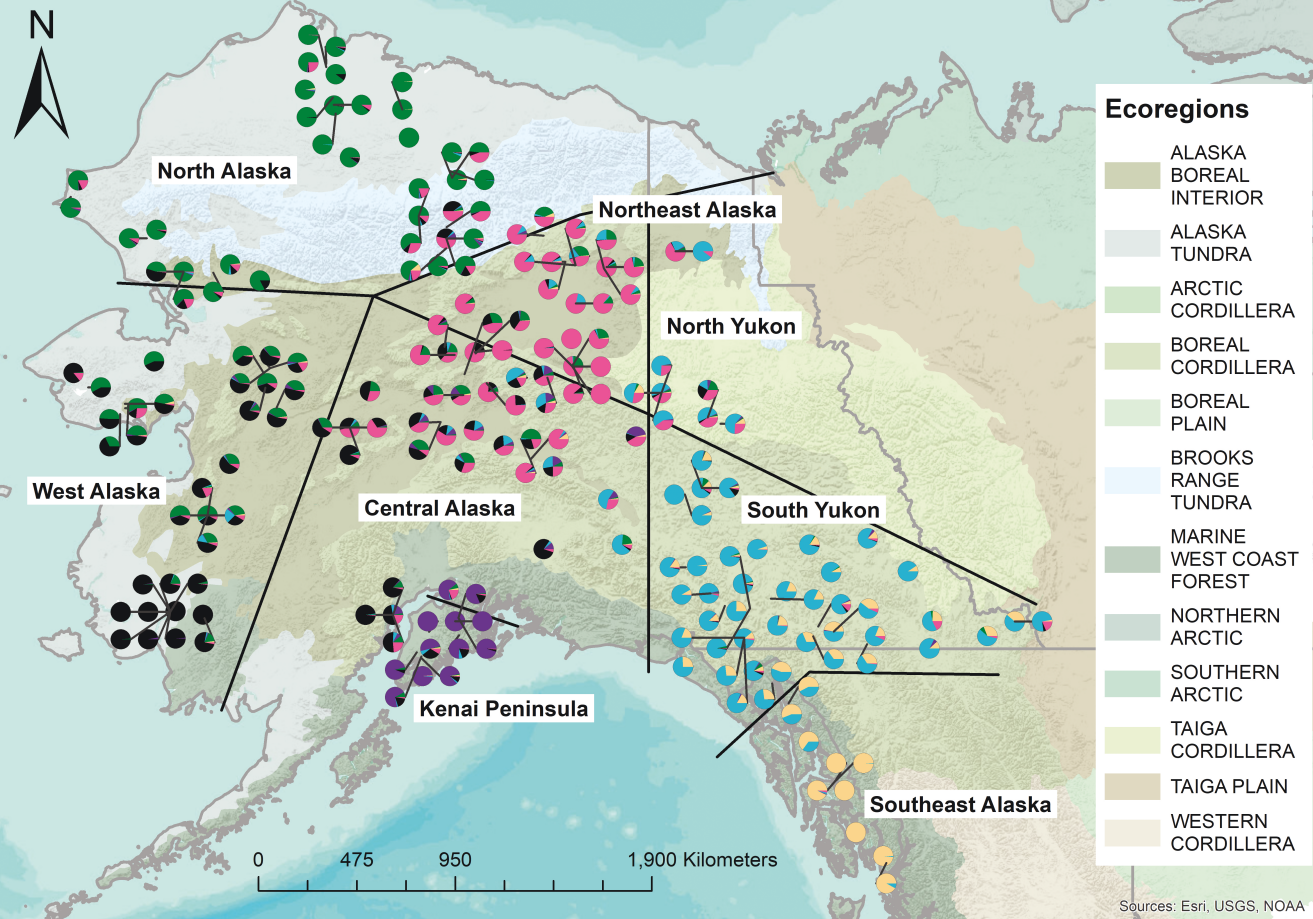
Alaska and Yukon Wolverine (*Gulo gulo*) genetic structure from the 201 single nucleotide polymorphism ( genotyped individuals. Pie charts represent *q*-values showing the proportion of each wolverine’s ancestry being assigned to 6 genetic clusters (statistically supported *K* value of *K* = 6). To display all pie charts without overlap, leader lines indicate sampling location for pie charts with location adjusted for visualization. Samples are grouped into 8 regional groups (North Alaska, West Alaska, Kenai Peninsula, Central Alaska, Northeast Alaska, North Yukon, South Yukon, and Southeast Alaska) denoted by black lines. Groupings were data informed by patterns of ancestry assignment from the SNP and microsatellite dataset, along with geographic features, including mountain ranges and the Kenai Peninsula, as well as ecoregion designations (see *Methods* section).

Eight regional groups were defined based on geographic and genetic data, including which ecoregions individuals were sampled from and geographic features and boundaries between regions (Fig. 1). Then, both microsatellite and SNP population structure results (see microsatellite and SNP results sections; Fig. 2) were used to determine regional groupings of samples. Broadly, the microsatellite and SNP data confirmed an east–west division, grouping samples from the Yukon and Southeast Alaska versus the rest of Alaska. Further divisions were made based on whether ancestry of an individual was majority assigned to 1 genetic cluster at the highest statistically supported *K* value (*K* = 6) in the SNP dataset (Fig. 3). Samples near each other and within the same ecoregion or geographic feature (mountain range, peninsula) and with the majority of their ancestry assigned to 1 genetic cluster were grouped. In some instances, where there was majority assignment to 1 genetic cluster but patterns of admixture different from most individuals in the genetic cluster, individuals were grouped with samples that had similar admixture patterns (e.g., 2 individuals grouped in the Central Alaska region majority assigned to South Yukon genetic cluster but with admixture patterns more similar to the Central Alaska region).

It is important to note that multiple individuals sampled in northern southeast Alaska were more strongly assigned to the South Yukon genetic cluster than the Southeast Alaska genetic cluster, and therefore northern southeast Alaska individuals are included in the South Yukon region. We had relatively few Wolverine samples representing southcentral Alaska, and although the majority of their ancestry was assigned to the West Alaska genetic cluster, their admixture patterns differed and therefore we grouped them in our Central Alaska region. We created a strict cut off grouping individuals on the Kenai Peninsula. One individual that was sampled near the peninsula was assigned to the Kenai Peninsula genetic cluster, but this sample was grouped in the Central Alaska region.

### Microsatellite results.

For our STRUCTURE analysis of the microsatellite dataset, the likelihood curve peaked at *K *= 3, with delta *K* selecting *K* = 3 (Supplementary Data SD6). In plots of q-values by individual, a high degree of admixture was observed across *K* clusters, with an east-west gradient at *K* = 2, and only Southeast Alaska showing some level of distinctiveness at *K* = 3 and 5 (Fig. 2a). PCA revealed an almost complete overlap of regional groups (Fig. 2c). Pairwise *F*_*ST*_ values for the microsatellite dataset ranged from 0.007 to 0.085, where the largest differentiation was observed between Southeast Alaska and the Kenai Peninsula (Table 1). The permutation test showed that 14 pairwise *F*_*ST*_ comparisons were significantly different from 0, rejecting the hypothesis of panmixia. Expected heterozygosity ranged from 0.519 on the Kenai Peninsula to 0.661 in south Yukon and Southeast Alaska (Table 2).

**Table 1.** Pairwise *F*_ST_ between regional Wolverine (Gulo gulo) sample groups sampled across Alaska and the Yukon, comparing the entire microsatellite dataset *F*_ST_ values (upper diagonal) and SNP dataset *F*_ST_ values (lower diagonal).

	N AK	W AK	KP	C AK	NE AK	N YK	S YK	SE AK
N AK	0.000	0.013	**0.049**	0.019	0.023	0.022	**0.023**	**0.061**
W AK	**0.012**	**0.000**	0.027	0.007	**0.035**	0.025	0.020	**0.057**
KP	**0.050**	**0.046**	**0.000**	**0.037**	**0.067**	**0.043**	**0.046**	**0.085**
C AK	**0.011**	**0.007**	**0.037**	0.000	0.010	0.011	0.012	**0.060**
NE AK	**0.016**	**0.017**	**0.051**	**0.009**	**0.000**	0.012	0.023	**0.074**
N YK	**0.019**	**0.019**	**0.050**	**0.011**	**0.013**	0.000	0.008	**0.057**
S YK	**0.026**	**0.027**	**0.051**	**0.017**	**0.021**	**0.009**	**0.000**	**0.038**
SE AK	**0.055**	**0.057**	**0.088**	**0.048**	**0.053**	**0.047**	**0.031**	0.000

Significant levels of differentiation from permutation tests between regions are bolded. All pairwise comparisons in the SNP dataset were significant. Samples regions are North Alaska (N AK), West Alaska (W AK), Kenai Peninsula (KP), Central Alaska (C AK), Northeast Alaska (NE AK), North Yukon (N YK), South Yukon (S YK), and Southeast Alaska (SE AK).

**Table 2.** Expected heterozygosity (*H*_*E*_) across regional Wolverine (Gulo gulo) groups sampled across Alaska and the Yukon, comparing the entire microsatellite dataset and SNP dataset.

Region	MSAT (*n*)	MSAT *H*_*E*_	MSAT *P*-value	SNP (*n*)	SNP *H*_*E*_	SNP *P*-value
Total	501	0.617		201	0.166	
N AK	96	0.583	**0.002**	38	0.169	0.042
W AK	43	0.606	0.014	33	0.169	0.050
KP	25	0.519	**0.002**	11	0.149	**0.002**
C AK	47	0.611	0.026	38	0.172	0.286
NE AK	29	0.625	0.192	20	0.168	0.084
N YK	40	0.636	0.702	9	0.158	0.012
S YK	187	0.652	0.008	42	0.167	**0.002**
SE AK	34	0.580	**0.002**	10	0.138	**0.002**

Sampled regions are North Alaska (N AK), West Alaska (W AK), Kenai Peninsula (KP), Central Alaska (C AK), Northeast Alaska (NE AK), North Yukon (N YK), South Yukon (S YK), and Southeast Alaska (SE AK). Bolded *P*-values indicate significantly lower region *H*_*E*_ compared to entire sample set at Bonferroni corrected alpha = 0.00625.

**Fig. 4.**
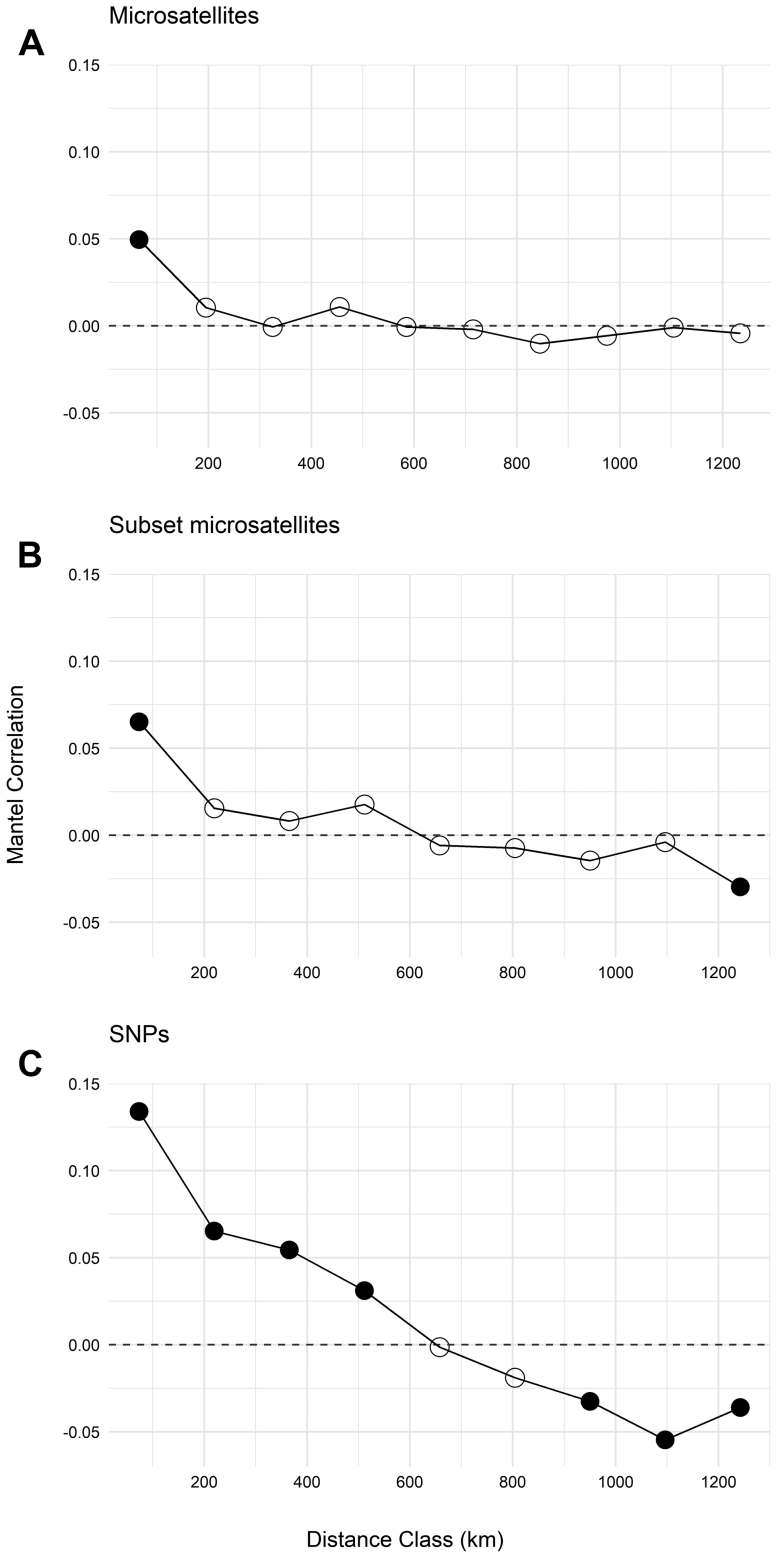
Spatial Mantel correlograms for wolverines (*Gulo gulo*) sampled across Alaska and the Yukon, comparing the entire microsatellite dataset (a, *n* = 492) subset microsatellite (b, *n* = 201) and SNP dataset (c, *n* = 201). Bins were determined using Sturges’ rule (a = 10 spatial bins, width 130 km; b and c = 9 spatial bins, width 146 km). Filled circles indicate Mantel *r* is significantly different from zero, and open circles indicate Mantel *r* values are not significantly different from zero following holm correction for multiple testing.

For our assessment of IBD, Euclidean genetic distance values for the full microsatellite dataset (*n* = 501) and the reduced subset microsatellite dataset (*n* = 201) were similar. There was a significant positive relationship between genetic and geographic distance for the full microsatellite dataset (*P* = 0.003) but small effect size (a low Mantel correlation, *r* = 0.043). The subset microsatellite dataset also showed a significant positive relationship (*P* = 0.010, *r* = 0.063). For the full microsatellite dataset, Mantel correlograms showed a positive autocorrelation in the first distance class (up to ~65 km; Fig. 4a) but did not show a point at which there was significant negative autocorrelation, while the subset microsatellite dataset showed a significantly positive autocorrelation in the first distance class (up to ~73 km) and significantly negative autocorrelation in the ninth distance class (~1,243 km, Fig. 4b).

### SNP results.

For our STRUCTURE analysis of the SNP dataset, the likelihood curve plateaued at *K* = 6 and delta *K* selected *K* = 2 (Supplementary Data SD6). At *K* = 2, an east-west gradient was observed but with less admixture per individual than the microsatellite dataset. Individuals in Southeast Alaska and the South Yukon were strongly assigned to the east genetic cluster, and individuals from North Alaska and West Alaska were strongly assigned to the west genetic cluster. Individuals from regions in between showed varying degrees of admixture but with higher assignment probabilities to the west genetic cluster (Fig. 2). At higher *K* values, individuals from the Kenai Peninsula were assigned to a distinct genetic cluster. At *K* = 6, clearer geographic patterns emerge with genetic clusters centered in North Alaska, West Alaska, Kenai Peninsula, Northeast and Central Alaska, North and South Yukon, and Southeast Alaska (Figs. 2 and 3). PCA revealed grouping within Kenai Peninsula, Southeast Alaska, and within South and North Yukon, while there was overlap of individuals from North, West, Central, and Northeast Alaska (Fig. 2c). Pairwise *F*_*ST*_ values for the SNP dataset ranged from 0.007 to 0.088, where the largest differentiation was observed between Southeast Alaska and the Kenai Peninsula (Table 1). All pairwise *F*_*ST*_ comparisons were significantly different than 0 for the SNP dataset. Expected heterozygosity ranged from 0.138 (Southeast Alaska) to 0.172 (Central Alaska; Table 1).

There was a significant positive relationship between genetic and geographic distance and a larger effect size for the SNP dataset (*P* = 0.0001, *r* = 0.185). The Mantel correlograms dataset showed significantly positive autocorrelation until the fourth distance class (up to ~512 km) and a negative autocorrelation starting at the seventh distance class (beyond ~950 km; Fig. 4c).

### Comparison of microsatellite and SNP results.

As predicted, the proportion ancestry assignment (*q*-values) per individual was greater across *K* values for the SNP dataset than the microsatellite dataset (Fig. 2). At *K* = 6, *q*-values showed almost complete individual assignment to different genetic clusters for Southeast Alaska and the Kenai Peninsula and revealed majority assignment to different genetic groups across North, West, and Northeast Alaska, and a genetic group in the Yukon. At *K* = 2 for the microsatellite dataset, a large degree of admixture was observed, and at higher *K* values most assignment proportions per individual were split across *K* values with Southeast Alaska being the only region to show distinctiveness.

The broad patterns for genetic differentiation and diversity were largely the same across datasets. The highest *F*_*ST*_ values observed for both datasets were between the Kenai Peninsula and Southeast Alaska, and patterns of *F*_*ST*_ were similar between data types. Permutation tests identified all pairwise *F*_*ST*_ comparisons as significantly different than 0 in the SNP dataset, while only 13 were identified as significant in the microsatellite dataset. Expected heterozygosity was significantly lower than the total for Southeast Alaska and the Kenai Peninsula for both datasets (Bonferroni corrected alpha of 0.00625), while only the microsatellite dataset identified North Alaska (*H*_*E*_ = 0.585) and only the SNP dataset identified the South Yukon (*H*_*E*_ = 0.167) as significantly lower than the dataset wide *H*_*E*_.

We observed a stronger IBD effect with the SNP dataset. Mantel tests for IBD resulted in greater significance and a Mantel *r* value for the SNP dataset (*P* = 0.0001, *r* = 0.185) almost 3 times greater than the reduced (*P* = 0.010, *r* = 0.063) and more than 4 times greater than the full microsatellite dataset (*P* = 0.003, *r* = 0.043). Mantel correlograms (Fig. 4) revealed a greater range of significant positive and negative autocorrelations for the SNP dataset than the genetic distance calculated from the reduced microsatellite dataset, and the SNP dataset revealed a significant but unexpectedly low positive correlation (*P *= 0.035, *r* = 0.074).

## Discussion

Population structure has been more clearly elucidated for highly vagile species with the transition to next-generation sequencing-based methods and genotyping with SNPs (Malenfant et al. 2015; Lah et al. 2016; Puckett and Eggert 2016). We compared population genetic analyses between the first SNP dataset generated for wolverines in North America and the largest microsatellite dataset generated for wolverines across Alaska and the Yukon. As we predicted, the SNP dataset detected greater population structure and a stronger signal of IBD, while estimating similar levels of diversity and differentiation despite having 40% of the samples in the microsatellite dataset. This is in line with previous studies across multiple fish species (Sunde et al. 2020) and for the Gunnison Sage-grouse (*Centrocercus minimus*; Zimmerman et al. 2020) where despite having 13% to 63% of the microsatellite sample size across studies, SNPs either detected the same or more population structure and similar levels of diversity and differentiation. In mammals with high dispersal capacity, SNPs have provided a pronounced increase in resolution of population structure via more separation of groups along PC axes and greater proportion of ancestry and higher supported *K* values in STRUCTURE analyses (Malennfant et al. 2015; Lah et al. 2016; Puckett and Eggert 2016).

Previous North America range-wide studies established distinct haplotypes and high differentiation between the southern and eastern peripheral populations and the northern populations (Kyle and Strobeck 2002; Zigouris et al. 2013), while studies focused within the northwestern range only detected isolation occurring on the Kenai Peninsula and in Southeast Alaska (Tomasik and Cook 2005; Krejsa et al. 2021). Our microsatellite and SNP datasets confirmed the genetic distinctiveness of wolverines in Southeast Alaska, while the SNP dataset supported a distinct group on the Kenai Peninsula and revealed additional genetic clusters grouping individuals from western Alaska, northern Alaska, northeastern Alaska, and the Yukon. Genetic clusters identified with the SNP dataset largely align with ecoregion designations and geographic features, suggesting that Wolverine gene flow across Alaska and the Yukon could follow an isolation by environment and/or an isolation by resistance model. Additionally, we confirmed our prediction that patterns of genetic differentiation and diversity would be relatively consistent between the datasets. This further demonstrates the value of genomic methods in uncovering previously undetected patterns of fine-scale population structure for highly vagile species like wolverines.

### Comparison of microsatellites and SNPs.

Both the SNP and microsatellite datasets revealed an east-to-west and a north-to-south population structure gradient, but the SNP dataset had higher supported *K* values and greater proportion assignment to genetic clusters at higher *K* values. A large amount of admixture was observed in our Central Alaska region from both data sets, suggesting gene flow from multiple populations into this region. A recent publication analyzing population structure of Eurasian wolverines with microsatellites also revealed comparable longitudinal structure in wolverines across Russia and Fennoscandia, with a high degree of admixture in the central regions (Bujnáková et al. 2024).

Our microsatellite analysis estimated a large degree of admixture among genetic clusters at supported *K* values from the STRUCTURE analyses, and only samples from Southeast Alaska showed evidence of genetic distinctiveness. Despite previous studies showing the Kenai Peninsula as a distinct population (Tomasik and Cook 2005; Krejsa et al. 2021), the region did not group strongly as a distinct genetic cluster when related individuals were removed from the dataset. However, at *K* = 3 with related individuals retained in the dataset, individuals from the Kenai Peninsula form a distinct group (Supplementary Data SD7 and SD8). Kresja et al. (2021) utilized 20 microsatellite loci to assess population structure across a similar distribution as our study, but with a larger proportion of samples from the Kenai Peninsula, Southeast Alaska, and the Yukon, and without samples from Southwest Alaska. Our microsatellite structure analysis revealed an eastern and a western divide first, while in Kresja et al. (2021), with related individuals removed, the Kenai Peninsula splits from the rest of Alaska and the Yukon first. This could be due to a larger proportion of Kenai Peninsula samples in their dataset because sample distribution can impact population clustering analyses (Kalinowski 2011). We also likely had lower power with our 12-microsatellite dataset because when we ran STRUCTURE and PCA on our subset microsatellite dataset, the optimal *K* value was *K* = 1 based on the likelihood curve and greater *K* values resulted in over-split *q*-values and even less definition of groups in PCA (Supplementary Data SD9, SD10 and SD14).

Our SNP dataset grouped the Kenai Peninsula as a genetic cluster at *K *= 3, supporting that it is a distinct genetic group, but contrary to Kresja et al. (2021) our SNP data suggest that South Yukon and Southeast Alaska are more distinct as they split into a genetic cluster at *K *= 2. The detection of subtle population structuring across Alaska and the Yukon provides new information on Wolverine connectivity across their northwestern range. The highest supported *K* value was *K* = 6, where the likelihood curve plateaus. Although *K* = 2 was best supported by delta *K* values, this method has been shown to over emphasize *K* = 2 (Janes et al. 2017; Stankiewicz et al. 2022). It has been recommended to evaluate log likelihood values up until the values plateau and until clusters are biologically meaningful (Pritchard and Wen 2002). The likelihood curve has also been shown to better evaluate structure under moderate levels of differentiation (Waples and Gaggiotti 2006). At *K* = 6, the SNP dataset revealed population groupings with biological significance as they largely aligned with ecoregions and geographic features. This structure was not detected with our microsatellite dataset and not detected with the 20-locus microsatellite dataset from Krejsa et al. (2021). What was previously thought to be a panmictic population aside from Southeast Alaska and the Kenai Peninsula could have underlying patterns of isolation by environment (Wang and Bradburd 2014) or isolation by resistance (McRae 2006) resulting in fine-scale population structuring.

Our regional estimates of low to high genetic diversity and differentiation were similar, but we detected some differences in patterns of *H*_*E*_ and *F*_*ST*_. Comparing *H*_*E*_ between the SNP and subset microsatellite dataset shows that differences can be attributed to sample size and distribution of samples for most regions (Supplementary Data SD11 and SD12). Nonetheless, the Kenai Peninsula remained the sampled region with the lowest diversity in our microsatellite dataset while Southeast Alaska was the sampled region with the lowest diversity in our SNP dataset. This result could be due to differences in microsatellite and SNP locus mutational mechanisms and histories (Morin et al. 2004). Significance of *F*_*ST*_ estimates differed largely between the SNP and microsatellite datasets, where only the larger *F*_*ST*_ values were significant for the microsatellite dataset, whereas all *F*_*ST*_ values were significant for the SNP dataset. Differing patterns of significance between the SNP and microsatellite datasets for *F*_*ST*_ and *H*_*E*_ values may be explained by the decrease in variance with an increased number of SNP loci (Waples 1998). For *H*_*E*_ significance calculations, variance was smaller for the SNP dataset (Supplementary Data SD11) and for *F*_*ST*_ significance calculations, confidence intervals were smaller for the SNP than the microsatellite dataset (Supplementary Data SD13).

Our IBD analysis revealed a significant relationship between genetic and geographic distance across all datasets, but the SNP dataset resulted in greater correlation and significance than the total and subset microsatellite datasets. Using microsatellites, Kresja et al. (2021) found that there was no significant correlation between individual pairwise genetic distance and geographic distance. Evaluations of IBD using populations (sites) as the unit of analysis demonstrated significant IBD in wolverines across Canada (Rico et al. 2015) and North America (Kyle and Strobeck 2001, 2002). Across the southern range of the species, Cegelski et al. (2006) did not detect significant IBD between sites—however, IBD was significant among individual wolverines across Idaho, Montana, and Wyoming (Balkenhol et al. 2020). Significant IBD was detected between individuals across all of Eurasia; however, comparisons within regions varied from high IBD detected in Fennoscandia to no IBD detected in the Eurasian Plains and Taymyr (Bujnáková et al. 2024). The differences in whether previous studies detected IBD could be attributed to whether their sample distributions spanned enough geographic distance to detect IBD for wolverines because their home range and dispersal distances are large. Furthermore, isolation by resistance or environment could be disrupting IBD patterns, or there was not enough power in the microsatellite datasets to calculate accurate genetic distances.

Our comparison of Euclidean genetic distance between the SNP and subset microsatellite datasets revealed an unexpectedly low positive correlation, suggesting the 12-locus microsatellite dataset may not precisely estimate individual genetic differences. To our knowledge, no direct comparison of genetic distance estimates between microsatellite and SNP datasets has been previously considered, but studies have shown SNPs improve precision over microsatellites in assigning individual black bears (*Ursus americanus*) to their natal population (Puckett and Eggert 2016) and increased accuracy in parentage assignment for European Bison (Wisent; *Bos bonasus*; Tokarska et al. 2009). Additionally, a recent study compared microsatellite and SNP datasets across multiple small mammal species and showed that genetic distance calculated from SNPs provided greater statistical power to determine isolation by resistance relationships (Skey et al. 2023).

Our spatial autocorrelation analysis utilizing the SNP dataset revealed higher correlation values and a larger number of significant distance classes than the total and subset microsatellite datasets. This same result for spatial autocorrelation has been shown in Pronghorn (*Antilocapra americana*), even though their SNP dataset did not describe more population structure than their microsatellite dataset (LaCava et al. 2020). Our SNP dataset resulted in multiple distance classes with significant positive (<512 km) and negative autocorrelation (>950 km). Across Idaho, Montana, and Wyoming, Balkenhol et al. (2020) revealed positive autocorrelation up to ~230 km and negative autocorrelation beyond distances of ~420 km. Our microsatellite and SNP datasets estimated larger distance classes at which negative autocorrelation is significant compared to results from Balkenhol et al. (2020) from the southern range. Our SNP results also revealed larger distance classes at which there is significant positive spatial autocorrelation compared to the southern range. Similarly, Kyle and Strobeck (2002) showed a higher positive correlation between genetic and geographic distance in the southern than the northern range. This pattern could be reflecting the more contiguous habitat in their northern range allowing for gene flow over greater distances.

### Population structure and ecoregions.

Our results show that genetic population structure in wolverines aligns with habitat-defined ecoregions, but with significant admixture along habitat boundaries (Fig. 3). The North Alaska genetic cluster was mostly present in Brooks Range tundra and Alaska tundra ecoregions. The West Alaska genetic cluster occupies largely marine west coast forest and Alaska tundra, while there was significant admixture between the West and the North genetic clusters in the Alaska boreal interior ecoregion and Alaska tundra on the Seward Peninsula. The West Alaska genetic cluster was also present in the central region in both the boreal and marine ecoregions, but with admixture from the Central and Northeast Alaska and Kenai Peninsula genetic clusters. The Northeast Alaska and Central Alaska genetic clusters align with Alaska boreal interior and boreal cordillera ecoregions, with varying degrees of admixture from the surrounding genetic clusters. The majority of the South Yukon genetic cluster aligns with boreal cordillera, and the North Yukon in taiga cordillera showed admixture between South Yukon and Northeast Alaska. Southern South Yukon in both marine and boreal ecoregions was admixed with the Southeast Alaska genetic cluster. The Kenai Peninsula genetic cluster and Southeast Alaska genetic cluster are characterized by the marine ecoregion, but also include significant geographic barriers likely resulting in the increased genetic differentiation and lower genetic diversity of these 2 regions.

We hypothesized that underlying natal-habitat-biased dispersal could be a mechanism for creating this subtle population structure in Wolverine populations. Our SNP population structure results generally supported our hypotheses that population structure would align with isolation by environment model because we detected genetic clusters aligned with coastal, boreal, and tundra ecoregions across Alaska and the Yukon. Natal-habitat-biased dispersal could be the underlying mechanism leading to ecoregions and genetic clusters largely aligning. There may also be isolation by resistance occurring, especially for the Kenai Peninsula and Southeast Alaska, where major landscape features such as mountain ranges and the peninsula are likely constricting gene flow. Previous studies have found population structure for carnivores inhabiting isolated versus mainland regions of Alaska (e.g., wolverines, Krejsa et al. 2021; brown bears, Morton et al. 2016; black bears, Robinson et al. 2007; wolves, Weckworth et al. 2005) while structure between regions without major geographic isolation in Alaska has only been shown in cervid populations, e.g., Caribou (*Rangifer tarandus*; Mager et al. 2014); Moose (*Alces alces*; Schmidt et al. 2009).

### Conservation and management implications.

Identifying geographic variation in genetic structure is an important precursor for informing the design of population units, which can aid in assigning conservation status and developing monitoring programs. Multiple Game Management Units in Alaska span each genetic cluster, and lower harvest limits and refugia from trapping largely align with more isolated regions. Our genetic results highlight the Kenai Peninsula and Southeast Alaska as the most genetically isolated and lowest diversity regions across our study area. Population assignment revealed 1 individual with majority ancestry assigned to the Kenai population was sampled just outside of the Kenai Peninsula, and an individual 1/3 assigned to the Kenai was sampled in the northeast corner of our Central Alaska region indicating migration out of the Kenai. Wolverines are managed for lower harvest levels and have harvest refugia near the base of the Kenai Peninsula; however, a detailed genetic monitoring framework would be beneficial in assessing dispersal rates in and out of the Kenai Peninsula to determine long-term maintenance of genetic diversity in this region.

The Yukon manages wolverines via Registered Trapping Concessions (RTCs), where there is no harvest quota for wolverines. Our population structure analyses indicate that gene flow between the Southern Yukon region and Southeast Alaska is moderate. Few RTCs are registered in the southwest corner of the Yukon (Kukka et al. 2022), which is largely comprised of Kluane National Park and Reserve. This region of low harvest pressure could serve as a harvest refugium and allow for the observed gene flow between Southeast Alaska and the Yukon. Additionally, our study did not assess any individuals from northern British Columbia. Previous analysis of population structure largely groups northern British Columbia individuals with individuals sampled from Southeast Alaska (Krejsa et al. 2021), indicating that we have not sampled the entire distribution of the population in our Southeast Alaska genetic cluster. A broader sampling of Southeast Alaska and northern and coastal British Columbia would be beneficial to assess the entire distribution of the population and patterns of gene flow across the mountain ranges bordering Southeast Alaska and further define gene flow between these areas and the Yukon.

Our detection of structure between the Northern, Western, and Central groups also raises questions about Wolverine connectivity in the face of increased human presence, natural resource development, and climate change. Arctic populations may be especially vulnerable to the effects of accelerated climate warming if Wolverine populations are structured due to natal-habitat-biased dispersal and either behaviorally prefer or are adapted to environmental characteristics of ecoregions (Hoban et al. 2016). One major advancement provided by SNP datasets is investigating signatures of selection in relation to environmental characteristics to understand patterns of local adaptation (Dauphin et al. 2023), which will be an area of future application of our RAD sequencing dataset.

The expansion of roads, natural resource extraction, and increasing human presence on the landscape could pose a threat to populations because these factors have impacted wolverines in the southern extent of their range (Scrafford et al. 2017; Heinemeyer et al. 2019; Sawaya et al. 2019; Balkenhol et al. 2020; Carroll et al. 2020). Proposed road and natural resource developments could create resistance features to Wolverine dispersal (Glass et al. 2022; Glass and Robards 2024). Our IBD results highlight that SNP datasets will likely be more sensitive to assessing isolation by resistance relationships with landscape features for wolverines in their northern range. Currently, monitoring for wolverines in Alaska or the Yukon consists largely of carcass collection-based health assessment and analysis of harvest statistics (Oakley et al. 2016; Jung et al. 2020; Kukka et al. 2022; Peraza et al. 2023; Chételat et al. 2024) as well as 1 recent collaring study on the North Slope of Alaska (Glass and Robards 2024). However, current monitoring can be biased by unreported harvest, and live-capture studies are limited by study area size and cost.

For long-term monitoring of population trends, genetic monitoring should be in place to assess the impacts of habitat changes in northern regions where there is subtle population structure and to monitor trends in genetic diversity for isolated regions. Our study serves as a baseline for further investigation into Wolverine gene flow in their core northwestern range and as the first genomic dataset for North American wolverines. We have demonstrated that transitioning to large SNP genetic datasets benefits the analysis of population structure and IBD for this highly vagile species. Carnivore species across their northern distributions serve as important cultural icons and subsistence resources to northern communities. Increasing conservation threats to northern carnivore populations from climate change and human development highlight the importance of utilizing the latest tools to assess populations and develop monitoring programs.

## Supplementary data

Supplementary data are available at *Journal of Mammalogy* online.

**Supplementary Data SD1.** North American distribution of wolverines. Map adapted from COSEWIC 2014. Original map produced by Bonnie Fournier, NWT.

**Supplementary Data SD2.** Wolverine (*Gulo gulo*) University of Alaska Museum of the North sample loan identifiers.

**Supplementary Data SD3.** Microsatellite loci, microsatellite PCR profile, and expanded outlier detection methods.

**Supplementary Data SD4.** Assessment of linkage disequilibrium, which was not evident at the region level or across total as no Benjamini–Hochberg (B-H) critical values were greater than the *P*-value.

**Supplementary Data SD5.** Assessment of Hardy–Weinberg equilibrium. Out of the 8 defined regions, locus Gg443 was out of Hardy–Weinberg equilibrium in South Yukon as indicated by the Benjamini–Hochberg (B-H) critical value being greater than the *P*-value.

**Supplementary Data SD6.** a) Alaska and Yukon Wolverine (*Gulo gulo*) microsatellite dataset program STRUCTURE mean likelihood and variance plot and Evanno plot per *K* value; and b) Wolverine SNP dataset likelihood and Evanno plots for optimal *K* value selection.

**Supplementary Data SD7.** Alaska and Yukon Wolverine (*Gulo gulo*) genetic structure plots from program STRUCTURE for the microsatellite dataset with related individuals kept in the analysis. *Q*-value plots show the proportion of each wolverine’s ancestry being assigned to different *K* values for 540 microsatellite genotyped samples. From left to right, samples are organized by region starting with North Alaska (N AK), West Alaska (W AK), Kenai Peninsula (KP), Central Alaska (C AK), Northeast Alaska (NE AK), North Yukon (N YK), South Yukon (S YK), and Southeast Alaska (SE AK).

**Supplementary Data SD8.** Alaska and Yukon Wolverine (*Gulo gulo*) microsatellite dataset with related individuals kept in program STRUCTURE mean likelihood and variance plot and Evanno plot per *K* value for optimal *K* value selection.

**Supplementary Data SD9.** Alaska and Yukon Wolverine (*Gulo gulo*) subset microsatellite dataset (*n* = 201) in program STRUCTURE mean likelihood and variance plot and Evanno plot per *K* value for optimal *K* value selection.

**Supplementary Data SD10.** Alaska and Yukon Wolverine (*Gulo gulo*) genetic structure plots from program STRUCTURE for the subset microsatellite dataset (*n* = 201). Each individual bar represents an individual Wolverine. From left to right, samples are organized by region starting with North Alaska (N AK), West Alaska (W AK), Kenai Peninsula (KP), Central Alaska (C AK), Northeast Alaska (NE AK), North Yukon (N YK), South Yukon (S YK), and Southeast Alaska (SE AK).

**Supplementary Data SD11.** Expected heterozygosity (*H*_E_) across regional Wolverine (*Gulo gulo*) groups sampled across Alaska and the Yukon, comparing the entire microsatellite dataset (µsat) and SNP dataset and subset microsatellite datasets (µsat subset). Sampled regions are North Alaska (N AK), West Alaska (W AK), Kenai Peninsula (KP), Central Alaska (C AK), Northeast Alaska (NE AK), North Yukon (N YK), South Yukon (S YK), and Southeast Alaska (SE AK). Bolded *P*-values (*P*-val) indicate significantly lower region *H*_E_ compared to entire sample set at Bonferroni corrected alpha = 0.00625, and variance (var) for significance calculations is shown.

**Supplementary Data SD12.** Pairwise FST between regional Wolverine (*Gulo gulo*) sample groups sampled across Alaska and the Yukon, using the entire (upper diagonal) and subset microsatellite (lower diagonal) dataset FST values. Significant levels of differentiation from permutation tests between regions are bolded. Samples regions are North Alaska (N AK), West Alaska (W AK), Kenai Peninsula (KP), Central Alaska (C AK), Northeast Alaska (NE AK), North Yukon (N YK), South Yukon (S YK), and Southeast Alaska (SE AK).

**Supplementary Data SD13.** Confidence intervals for microsatellite (µsat), SNP, and subset microsatellite (µsat sub) dataset’s *F*_ST_ significance calculations. Samples regions are North Alaska (N AK), West Alaska (W AK), Kenai Peninsula (KP), Central Alaska (C AK), Northeast Alaska (NE AK), North Yukon (N YK), South Yukon (S YK), and Southeast Alaska (SE AK).

**Supplementary Data SD14.** Alaska and Yukon Wolverine (*Gulo gulo*) genetic structure from PCA for the subset microsatellite dataset (*n* = 201).

**Supplementary Data SD15.** Distribution of relatedness estimates for SNPs using the Wang estimate of relatedness (left) and microsatellites using maximum-likelihood relatedness (right).

gyae151_suppl_Supplementary_Data_SD1

gyae151_suppl_Supplementary_Data_SD2

gyae151_suppl_Supplementary_Data_SD3

gyae151_suppl_Supplementary_Data_SD4

gyae151_suppl_Supplementary_Data_SD5

gyae151_suppl_Supplementary_Data_SD6

gyae151_suppl_Supplementary_Data_SD7

gyae151_suppl_Supplementary_Data_SD8

gyae151_suppl_Supplementary_Data_SD9

gyae151_suppl_Supplementary_Data_SD10

gyae151_suppl_Supplementary_Data_SD11

gyae151_suppl_Supplementary_Data_SD12

gyae151_suppl_Supplementary_Data_SD13

gyae151_suppl_Supplementary_Data_SD14

gyae151_suppl_Supplementary_Data_SD15

gyae151_suppl_Supplementary_Material

## Data Availability

Dryad DOI: 10.5061/dryad.crjdfn3dzC. Code available at https://github.com/elisestacy
