# Supplementary figures and images for: Comparing microsatellites and single nucleotide polymorphisms to evaluate genetic structure and diversity in wolverines (Gulo gulo) across Alaska and western Canada
Source: J Mammal. 2025 Jan 15;106(3):561–75. doi: 10.1093/jmammal/gyae151 (PMC12159531; doi:10.1093/jmammal/gyae151)

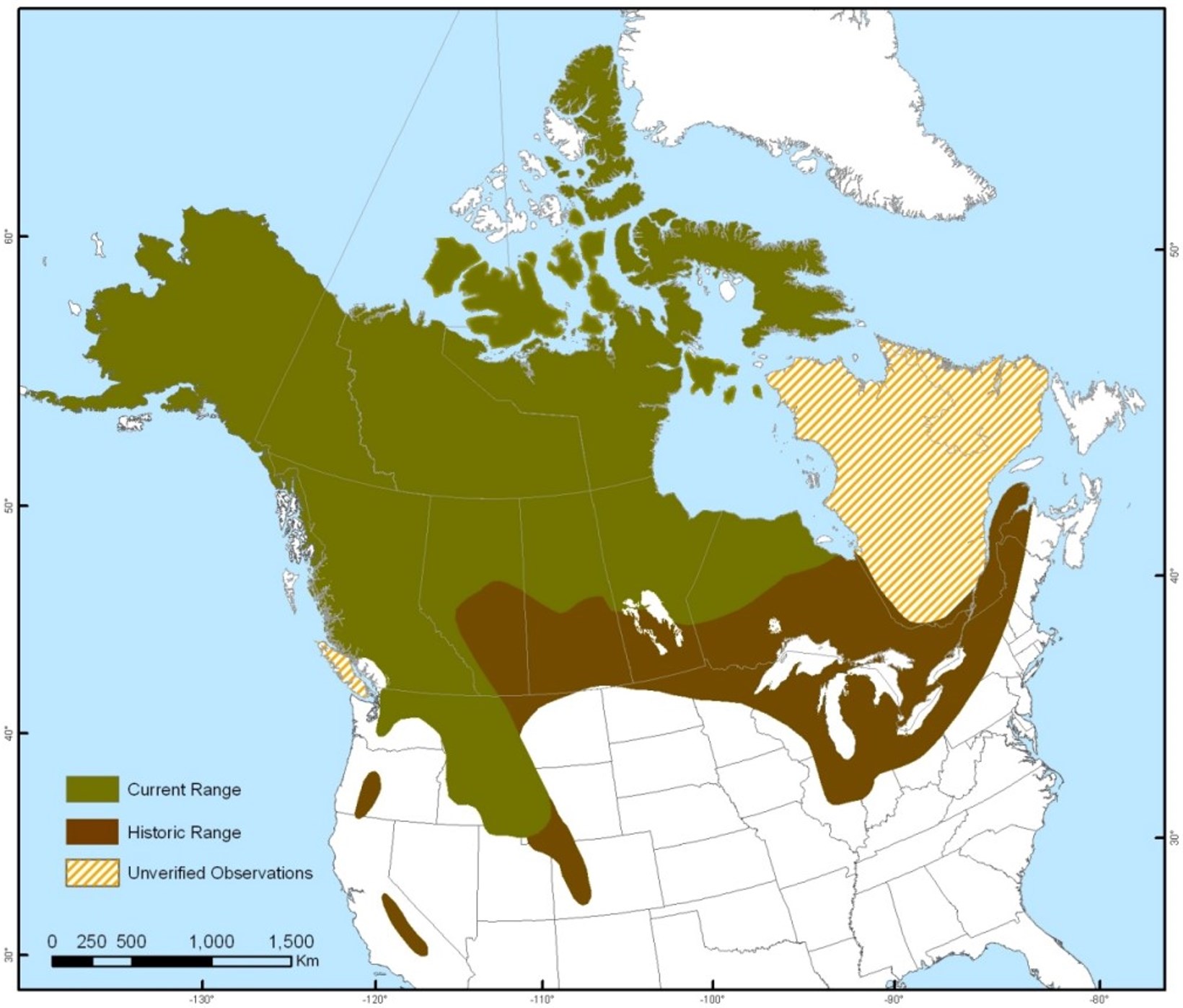

Supplement: gyae151_suppl_Supplementary_Data_SD1 [file gyae151_suppl_supplementary_data_sd1.jpeg]

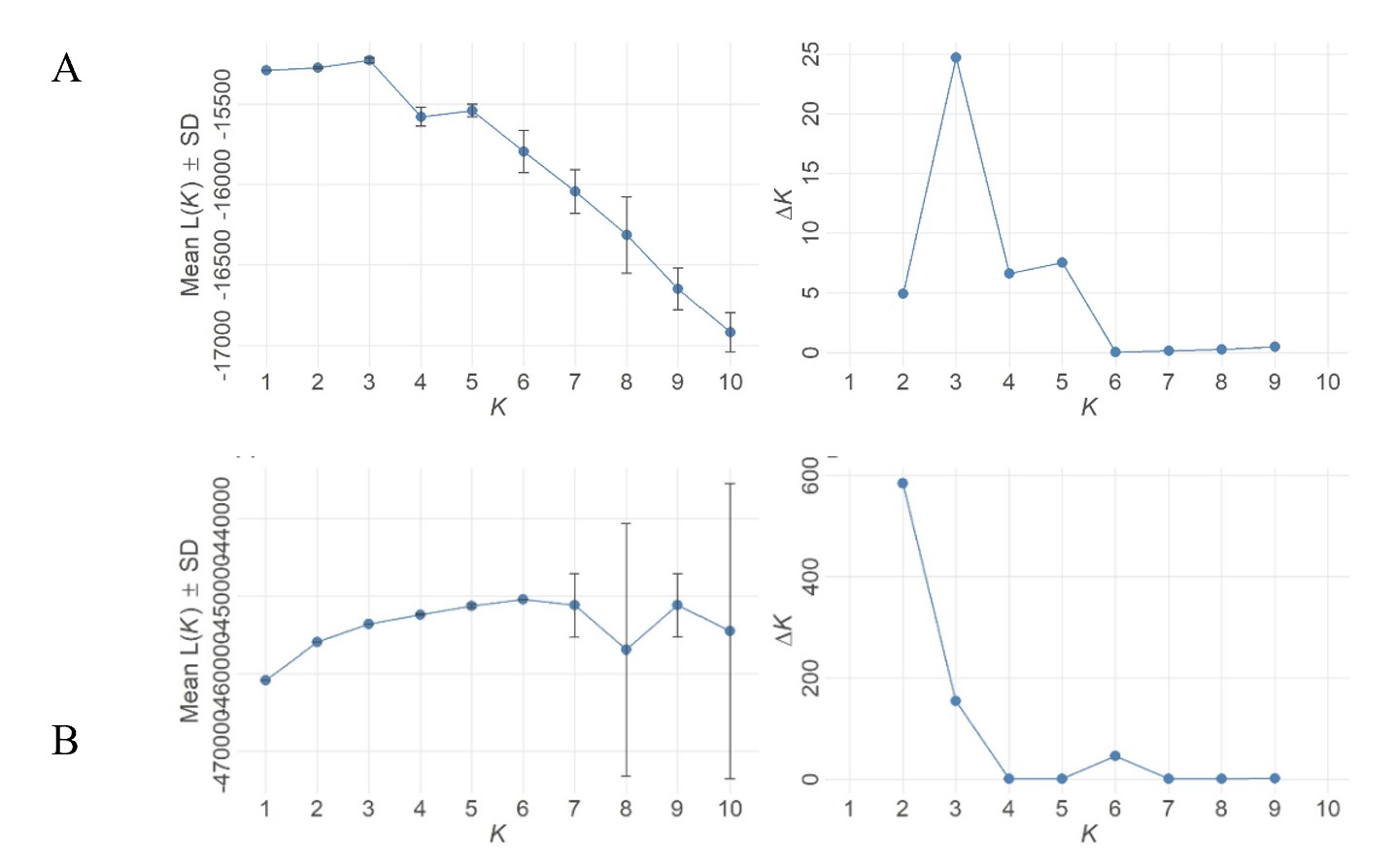

Supplement: gyae151_suppl_Supplementary_Data_SD6 [file gyae151_suppl_supplementary_data_sd6.jpeg]

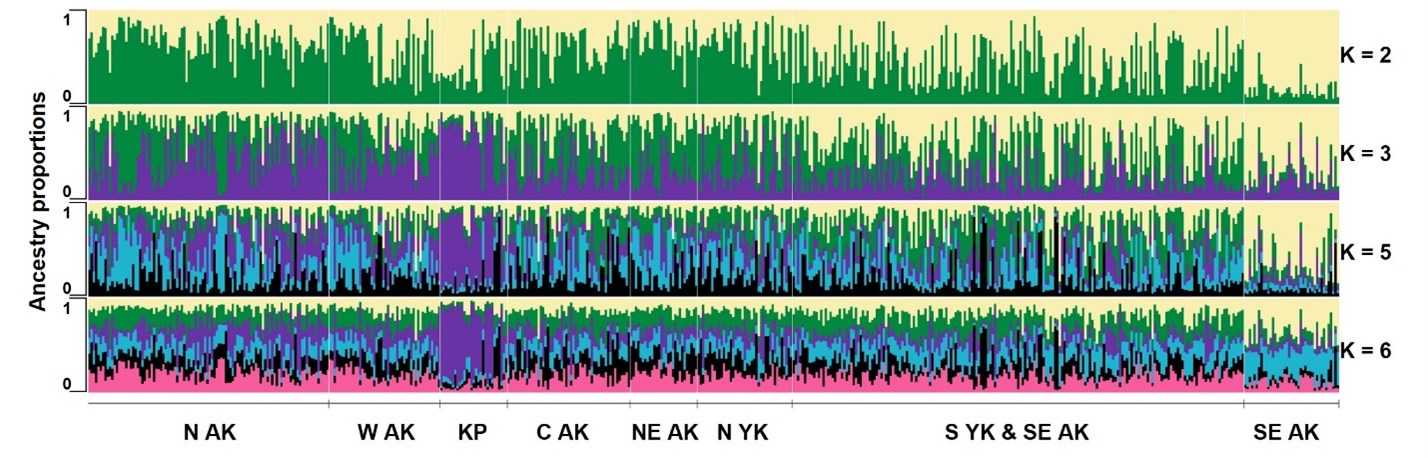

Supplement: gyae151_suppl_Supplementary_Data_SD7 [file gyae151_suppl_supplementary_data_sd7.jpeg]

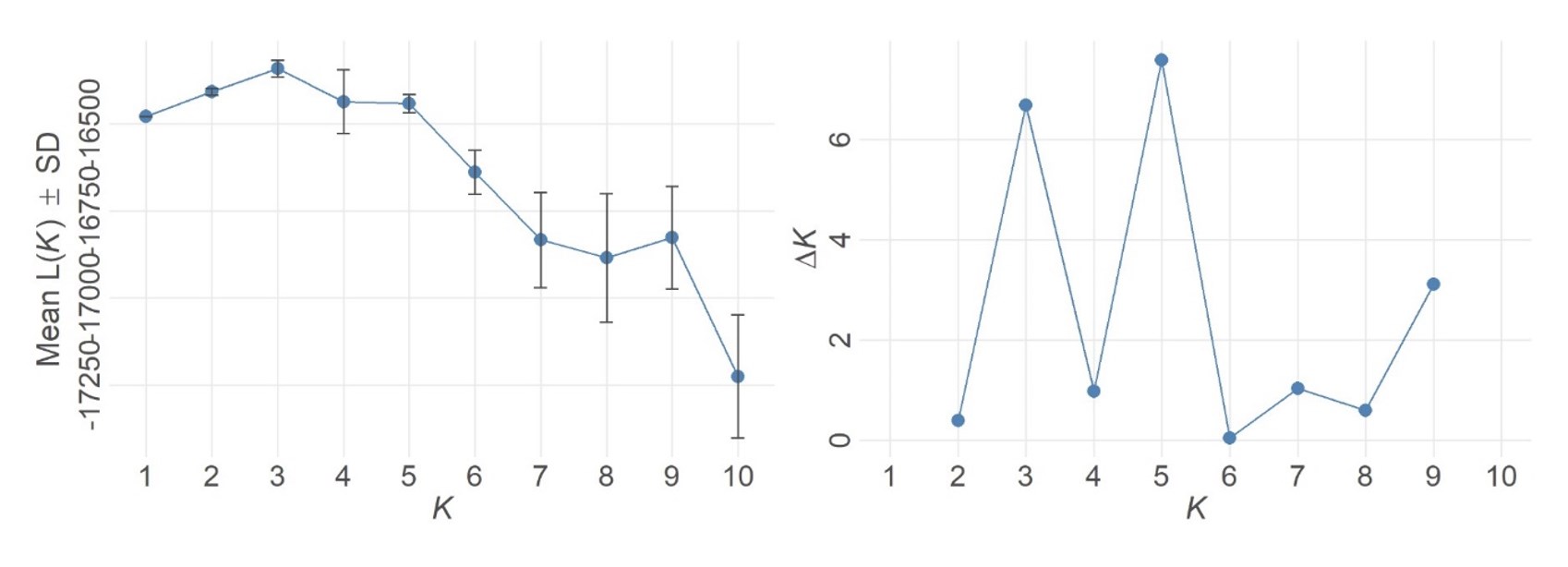

Supplement: gyae151_suppl_Supplementary_Data_SD8 [file gyae151_suppl_supplementary_data_sd8.jpeg]

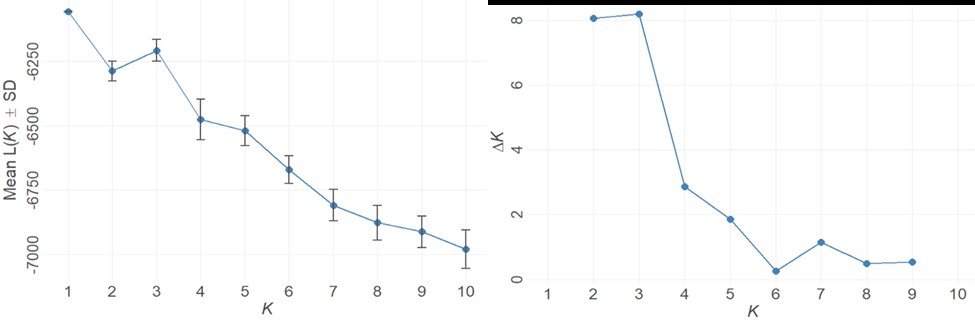

Supplement: gyae151_suppl_Supplementary_Data_SD9 [file gyae151_suppl_supplementary_data_sd9.jpeg]

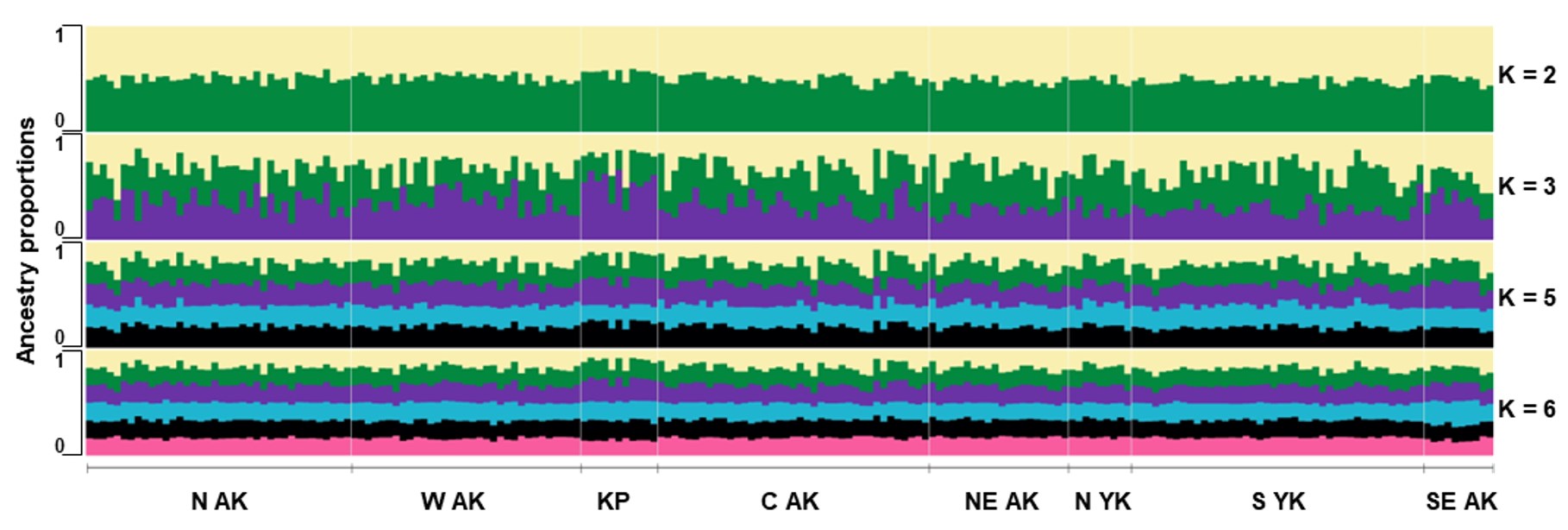

Supplement: gyae151_suppl_Supplementary_Data_SD10 [file gyae151_suppl_supplementary_data_sd10.jpeg]

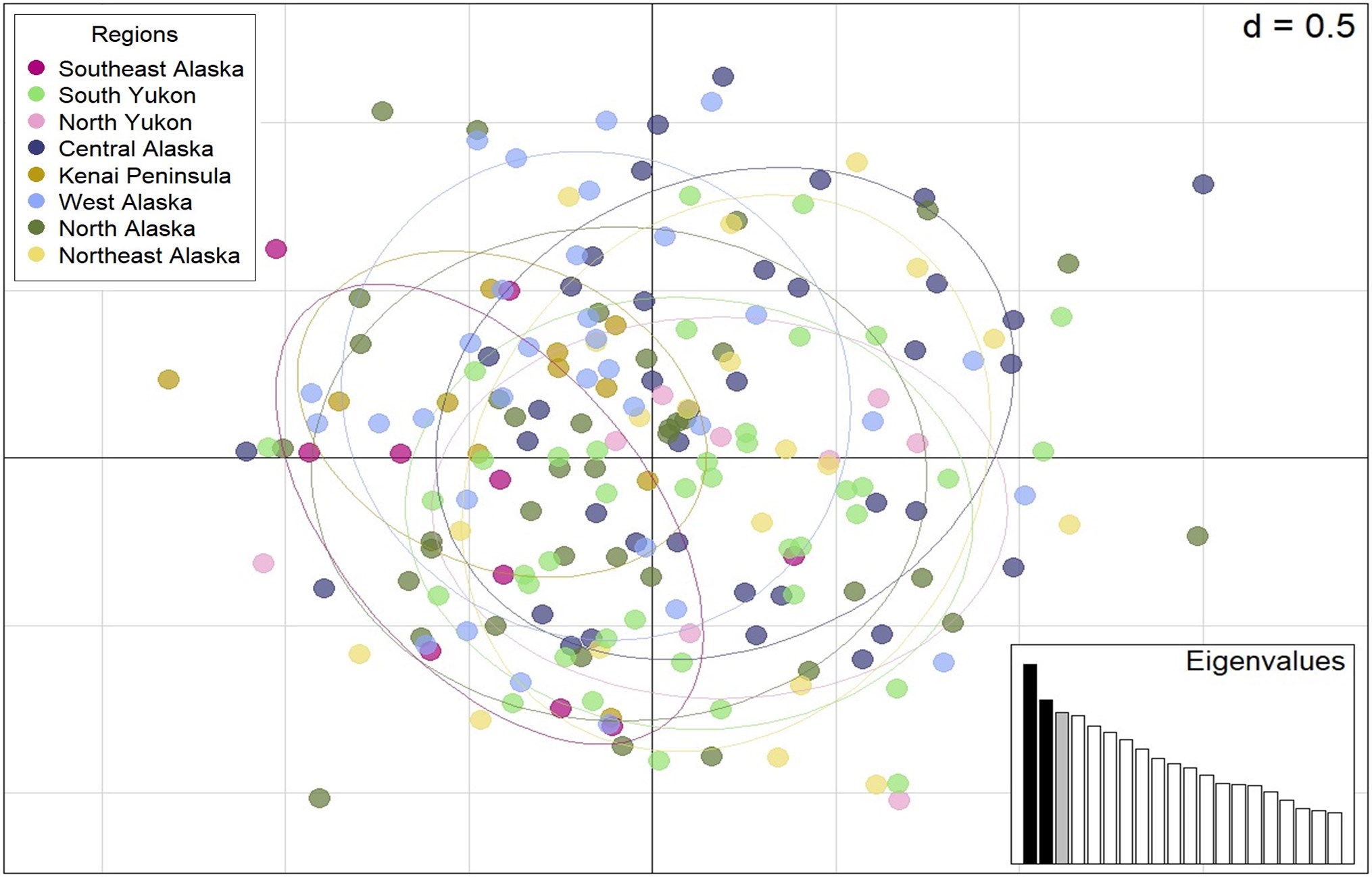

Supplement: gyae151_suppl_Supplementary_Data_SD14 [file gyae151_suppl_supplementary_data_sd14.jpeg]

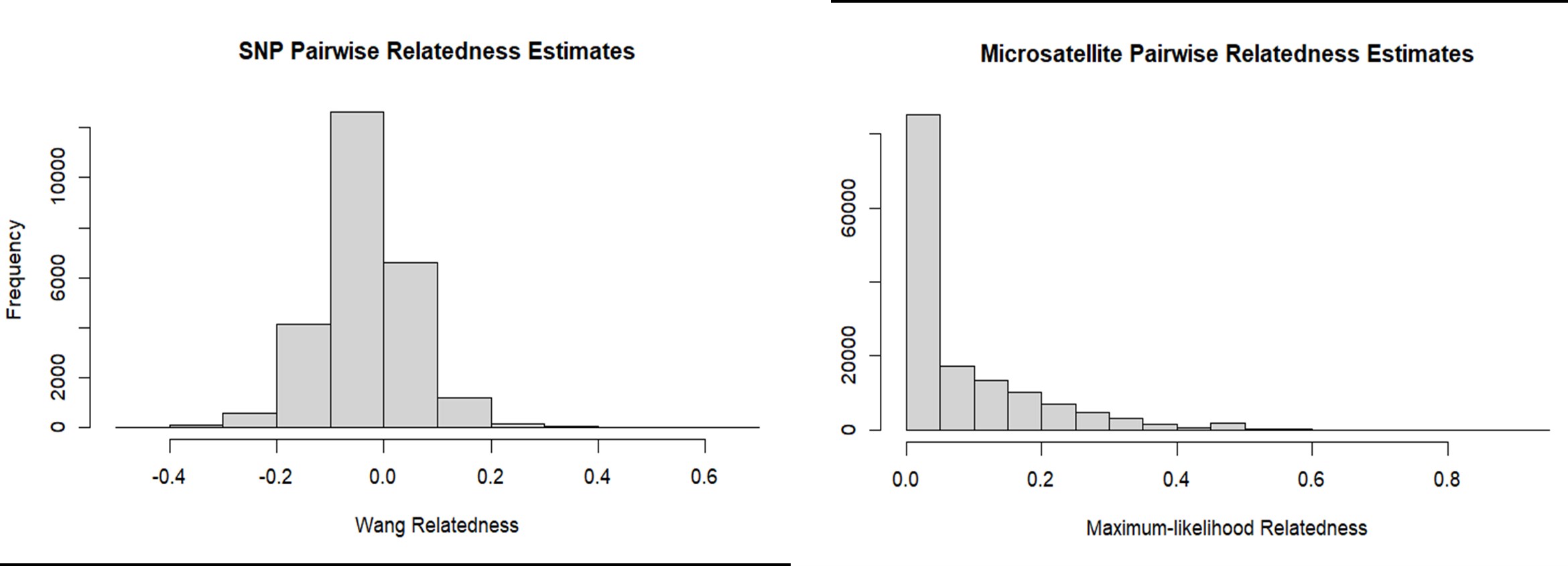

Supplement: gyae151_suppl_Supplementary_Data_SD15 [file gyae151_suppl_supplementary_data_sd15.jpeg]
