# Supplementary material for: Comparing microsatellites and single nucleotide polymorphisms to evaluate genetic structure and diversity in wolverines (Gulo gulo) across Alaska and western Canada
Source: J Mammal. 2025 Jan 15;106(3):561–75. doi: 10.1093/jmammal/gyae151 (PMC12159531; doi:10.1093/jmammal/gyae151)
Supplement: gyae151_suppl_Supplementary_Data_SD3 [file gyae151_suppl_supplementary_data_sd3.docx]

**Microsatellite loci and PCR profile:** Wolverines were genotyped in duplicate at 12 polymorphic microsatellite loci (Gg454, Gg443, Gg452, Gg465 (Walker et al., 2001) Gg42-1, Gg192-1, Gg37-2 (Walker et al., 2001, redesigned Krejsa et al., 2021), Gg3, Gg14, Tt-4 (Davis & Strobeck, 1998), Gg-7-1 (Davis & Strobeck, 1998, redesigned Krejsa et al., 2021), Ggu216 (Duffy et al., 1998)) in a single PCR multiplex with DNA extraction and PCR negative controls. The 7-µl PCR reaction contained 0.33 µM Gg454, 0.17 µM Gg192_1, 0.13 µM Gg3, 0.10 µM Ggu216, 0.09 µM Gg 443, 0.09 µM Gg452, 0.06 µM Gg42_1, 0.06 µM Gg465, 0.06 µM Tt-4, 0.04 µM Gg14, 0.04 µM Gg7_1, 0.03 µM Gg37_2, 3.5-µl of 1x concentration Qiagen Master Mix, 0.7-µl of 0.5x concentration Qiagen Q solution, and 1-µl DNA extract. The PCR profile had an initial 15 min denaturation of 94℃, a touchdown of 17 cycles of 30 s at 94℃, 90 s at 62℃ with a decrease in annealing temperature of 0.4℃ in each cycle, 1 min at 72℃, then 23 cycles starting with 30 s at 94℃, 90 s at 55℃ annealing, 1 min at 72℃, and a final extension for 30 min at 60℃. PCR products were separated using an Applied Biosystems 3130xl capillary machine (Applied Biosystems Inc., Foster City, CA, USA), with GeneScan 500 LIZ (Applied Biosystems Inc.) size standard and genotypes were scored with GENEMAPPER 5.0 (Applied Biosystems Inc.).

**Outlier detection methods:** We ran three outlier tests on the quality filtered and thinned SNP to detect loci potentially under selection. We used the function pcadapt from the R package “*pcadapt*” (Luu et al. 2017) which uses principal component analysis to estimate population structure and assumes that loci are outliers with respect to how they are related to structure. The computed p-values test for outliers and the test is based on the correlations between genetic variation and the first K principal components. We used the function tess3 from the R package “*tess3r*” (Caye et al. 2016) to estimate structure and detect outlier loci based on allele frequency differentiation tests. Tess3 utilizes genotype and geographic sample location to estimate structure with matrix factorization algorithms. We tested *K* = 1:10 with 10 repetitions each and used the pvalue function in “*tess3r*” on the *K* = 6 estimates to detect outliers. We used the snmf function in R package “*LEA*” (Frichot and François 2015) to estimate structure and detect outlier loci with methods similar to “*tess3r*” but without geographic sample information. We tested *K* = 1:10 with 10 repetitions each and used the function snmf.pvalues in “*LEA*” on the *K* = 6 estimates to detect outliers. For all three tests, we selected outlier loci based on an alpha value of 0.05 after correcting the p-values for multiple comparisons with a Benjamini-Hochberg Procedure using the function p.adjust in the “*stats*” R package.
